# Factors related to heart rate variability among firefighters

**DOI:** 10.1186/s40557-016-0111-6

**Published:** 2016-06-13

**Authors:** Jae-Hong Shin, Jung-Youb Lee, Seon-Hee Yang, Mi-Young Lee, In-Sung Chung

**Affiliations:** Division of Occupational and Environmental Medicine, Keimyung University School of Medicine, Daegu, South Korea; Division of Occupational and Environmental Medicine, Department of Preventive Medicine, Keimyung University School of Medicine, Daegu, South Korea

**Keywords:** Heart rate variability, Risk factors, Firefighters

## Abstract

**Objectives:**

The aim of this study was to investigate factors associated with heart rate variability in firefighters working in a metropolitan city in South Korea.

**Methods:**

Self-administered questionnaires including Korean Occupational Stress Scale (KOSS) as well as surveys collecting socio-demographic characteristics and work-related factors were given to 962 firefighters. After exclusion for missing data, 645 firefighters were included, and analysis of covaiance adjusted for the general risk factors and job characteristics were used to assess the relationship between heart rate variability and associated factors.

**Results:**

SDNN and RMSSD and were decreased in the area of occupational climate of the group with high job stress (*p* = 0.027, *p* = 0.036). HF(ln) was decreased in the area of organizational system and occupational climate of the group with high stress that statistically significant level (*p* = 0.034, *p* = 0.043).

**Conclusions:**

Occupational climate and organizational system are associated with reduction of heart rate variability. Preventive medical care plans for cardiovascular disease of firefighters through the analysis and evaluation of job stress factors are needed.

## Background

Firefighters are exposed to dangerous environment, emergency situations and irregular working hours due to natures of their work. They do not only extinguish a fire, but also need to do other works such as rescue of a life in disaster situations, emergency medical services and administrative tasks [1, 2]. The working environment causes physical and mental stresses, which give various diseases such as musculoskeletal disorders and sleep disorders [3]. Among them, several studies have revealed that job stress occurring in high tension situations is highly associated with occurrence of cardiovascular diseases [4–8]. It has been reported that workers in high tension situations have more risk factors of cardiovascular diseases [9, 10]. According to several studies of firefighters in the United States, the incidence of cardiovascular diseases tends to increase [11]. Sudden Cardiac Death (SCD) including myocardial infarction or arrhythmia, and traffic accidents in emergency situations accounted for the largest proportion of deaths of volunteer firefighters in the US between 1994 and 2004 [12]. Cardiovascular diseases accounted for 45 % of the total causes of death of firefighters. It is much higher than 22 % in police officers, 11 % in personnel working in emergency medical service and 15 % in workers in general industries [13]. According to the study targeting American firefighters who were died of sudden cardiac death under the age of 45 from 1996 to 2012, the firefighters engaged in emergency situations had higher risks of sudden cardiac death than those who worked in tasks not associated with emergency situations [14].

Heart rate variability represents the periodic variation of heart rate over time and it has the clinical meaning as the tool to predict the risk of recurrence of sudden cardiac death and arrhythmias such as atrial fibrillation in patients diagnosed with myocardial infarction [15, 16]. Moreover, several studies have demonstrated that it is significant as the predictive index of heart diseases in the general population [17]. According to recent studies, the stress causes overactivity of the sympathetic nervous system, which decreases the interaction of the autonomic nervous system controlling the variation of the heart rate. Thus, such a reduction or imbalance of the heart rate variability may cause heart diseases [18, 19]. However, there are almost no studies on the relationship between job stress and heart rate variability in the group of firefighters with high job stress.

For this reason, purpose of this study is to assess the relationship between job stress and heart rate variability among general characteristics affecting the heart rate variability and job-related factors in firefighters. In addition, we analyze the factors of cardiovascular diseases and use the results as the evidence for the program to prevent the cardiovascular diseases in this study.

## Methods

### Study population

We conducted survey and clinical examination targeting 962 firefighters in four fire stations located in the metropolitan city that visited the general hospital in order to take health exam from November 12^th^ 2012 to December 17^th^ 2012. The following subjects were excluded as follows: 232 subjects had problems in measurement of heart rate variability or had incomplete answers in questionnaires. Some subjects suffered from the following diseases which could affect heart rate variability such as 49 subjects with hypertension or diabetes mellitus, 6 subjects with arrhythmia, 4 subjects with myocardial infarction, angina and other ischemic heart disease. Lastly, 26 female firefighters excluded from this study. Thus, we conducted the analysis targeting 645 subjects.

### Variables

#### General and occupational characteristics

We conducted the survey using a standardized self-administered questionnaire. A doctor reviewed the answered questionnaires during the medical consultation and corrected them if missing or incorrect answers were found. The questionnaire consisted of general characteristics, job-related characteristics and job stress factors. General characteristics consisted of age, marital status, smoking status, drinking status, exercise status and body mass index (BMI). For smoking status, subjects were divided into the group of currently smoking and the group of currently not smoking. For drinking status, subjects were divided into the group of almost or never drinking alcohol and the group of the rest people. For exercise status, subjects were divided into the group of doing exercise producing sweats for 30 min 3-4 times in a week and the group of the rest people. For job-related characteristics, subjects were divided into groups by departments and shift work. According to fire officers Act article 14, jobs were divided into four kinds of jobs such as Fire extingish, rescue, emergency medical service and administration. Fire extingish, rescue and emergency medical services were assigned as on-site jobs. Administration was assigned as the office job. Shift work was divided into the group of non-shift and the group of 24-h shifts or 3 shifts. Items for medical history and current medications included in the questionnaire were re-confirmed and corrected during the medical consultation.

#### Korean occupational stress scale (KOSS)

Job stress factors were analyzed by using the Korean Occupational Stress Scale (KOSS) [20]. KOSS consisted of a total of 43 questions including the 8 subscales: difficult physical environment, high job demand, insufficient job control, inadequate social support, job insecurity, organizational system, lack of reward, and discomfort in the occupational climate. Score of each area was converted into 100 points, and total score of job stress was calculated by dividing a total score of all 8 areas by 8. Once a median was calculated in 8 sub-areas and a total score, groups were divided into the group with high job stress and the group with low job stress on the basis of the median.

#### Heart rate variability (HRV)

Heart rate variability was measured by using SA-3000P (Medicore, 2012). Once subjects sat in a chair, the electrodes were placed on the left and right wrists, and left ankle. After that, measuring heart rate for 5 min. Subjects were in a relaxed state and stared at the front opening their eyes at the same time in order to control changes in heart rate variability caused by the experimental environment upon measurement. It was measured in a place with bright lighting and no noise from the outside. The heart rate variability was analyzed in time domain and frequency domain, respectively. For time domain, we measured SDNN (the standard deviation of the NN interval) representing the entire variability through variation from the mean as the standard deviation of entire RR interval, and RMSSD (the square root of the mean squared differences of successive NN interval) which could assess the activities of the parasympathetic nervous system among the autonomic nervous system involved in the heart. For frequency domain, we measured LF (the low frequency component) representing both activities of the sympathetic and parasympathetic nervous systems with the frequency range of 0.04 ~ 0.15Hz and being mainly used as the activity index of the sympathetic nervous system, HF (the high frequency component) representing the activities of the parasympathetic nervous system (mainly vagus nerve), and LF/HF ratio reflecting the balance between sympathetic nervous system and parasympathetic nervous system as it was proportional to activities of the sympathetic nervous system but it was inversely proportional to activities of the parasympathetic nervous system. Among them, as the values of LF and HF in the frequency domain shows positively skewed distribution, we used log-transformed LF(ln) and HF(ln) [21].

### Statistical analysis

Among general characteristics of the subjects, status of smoking, drinking and marriage, shift work, exercise status, obesity, departments, KOSS total scores and relationships between items of sub-areas and heart rate variability were analyzed by using independent *T*-test. Analysis of variance was performed in the relationships between age and heart rate variability. Based on these results, groups were divided on the basis of converting a total score of KOSS survey and scores of each sub-area to analyze the effects of job stress on the heart rate variability. The groups and each item of the heart rate variability were assigned as the independent variable and dependent variable, respectively. We used analysis of covariance (ANCOVA) corresponding to the univariate general linear model. And age, status of smoking, drinking and marriage, shift work, departments which affected the heart rate variability were assigned as the covariance to control their effects. All statistical analyses were performed by using SPSS statistical software package (Version 19.0, IBM Corp., Armonk, NY, USA). For all analyses, significance levels were p-value < 0.05.

## Results

A total of 645 subjects were used in this study. The age of subjects ranged from 20s to 50s (20s, *n* = 58 (9.0 %); 30s, *n* = 234 (36.3 %); 40s, *n* = 272 (42.2 %); 50s, *n* = 81 (12.6 %)). Subjects were divided by departments as follows; 336 (52.1 %) in Fire extingish, 62 (9.6 %) in rescue, 111 (17.2 %) in emergency medical services and 136 (21.1 %) in administration. When it was applied to on-site jobs (Fire extingish, rescue and emergency medical services) and office job (administration), subjects were categorized as follows: 509 (78.9 %) in on-site jobs and 136 (21.1 %) in office job.

For smoking, 514 subjects (79.7 %) did not smoke and 131 subjects (20.3 %) were currently smoking. 231 subjects (35.8 %) almost did not or not drink. 303 subjects (47.0 %) did exercise producing sweats for 30 min 3 times in a week. 259 subjects (40.2 %) were obese with body mass index (BMI) of 25 or higher (Table 1).

**Table 1:** General and job characteristics of study subjects

Variables	Number	Percent
Age (year)		
20-29	58	9.0
30-39	234	36.3
40-49	272	42.2
50-59	81	12.6
Department		
Non-administrative job		
Fire extinguish	336	52.1
Rescue	62	9.6
Emergency medical service	111	17.2
Administrative job		
Administration	136	21.1
Shift work		
No	120	18.6
Yes	525	81.4
Smoking		
No	514	79.7
Yes	131	20.3
Alcohol		
No	231	35.8
Yes	414	64.2
Marital status		
Unmarried	123	19.1
Married	522	80.9
Exercise (times/week)		
< 3	342	53.0
≥ 3	303	47.0
BMI		
< 25	386	59.8
≥ 25	259	40.2

According to the analysis on the heart rate variability associated with demographic characteristics and job-related characteristics, the heart rate variability was decreased (*p* < 0.001) with increasing age in SDNN, RMSSD, LF(ln) and HF(ln) except LF/HF ratio. According to the analysis on the heart rate variability associated with smoking status, drinking status, marital status, shift work and departments, LF(ln) was increased in the group of smoking (*p* = 0.043). SDNN, RMSSD and LF(ln) were increased in the group of drinking (*p* = 0.004, *p* = 0.031, *p* < 0.001). RMSSD was decreased in the group of the office job and non-shift compared to that in the group of on-site jobs and shift work (*p* = 0.004, *p* < 0.001). In marital status, all indices of heart rate variability except LF/HF ratio were decreased in the married group (Table 2).

**Table 2:** Mean values of heart rate variability by general characteristics

					Unit; Mean (S.D.)
	Time domain		Frequency domain		
Variables	SDNN	RMSSD	LF(ln)	HF(ln)	LF/HF ratio
Age (year)					
20-29	45.63 (14.94)*	39.41 (19.80)*	6.02 (0.75)*	5.71 (0.96)*	1.94 (1.74)
30-39	42.72 (15.41)	33.64 (16.43)	5.90 (0.95)	5.40 (1.05)	2.72 (3.59)
40-49	34.89 (12.50)	26.03 (14.05)	5.34 (0.90)	4.80 (1.07)	2.66 (3.43)
50-59	27.64 (11.05)	19.66 (7.73)	4.77 (0.92)	4.35 (0.95)	2.34 (2.38)
Department					
Administrative job	36.49 (12.43)	26.54 (11.60)*	5.54 (0.88)	5.01 (0.92)	2.60 (2.92)
Non-Administrative job	38.50 (15.18)	30.11 (16.54)	5.59 (0.99)	5.07 (1.14)	2.66 (3.49)
Shift work					
No	35.87 (12.79)	25.78 (11.11)*	5.50 (0.93)	4.96 (0.91)	2.39 (2.30)
Yes	38.58 (15.01)	30.17 (16.46)	5.61 (0.97)	5.08 (1.13)	2.70 (3.57)
Smoking					
No	37.62 (14.79)	28.69 (15.31)*	5.54 (0.98)*	5.02 (1.10)	2.63 (3.18)
Yes	39.87 (14.03)	31.96 (16.92)	5.73 (0.91)	5.21 (1.07)	2.71 (4.05)
Alcohol					
No	35.87 (13.28)*	27.66 (13.75)*	5.39 (0.96)*	4.94 (1.04)	2.40 (3.39)
Yes	39.31 (15.24)	30.30 (16.62)	5.68 (0.95)	5.12 (1.12)	2.78 (3.36)
Marital status					
Unmarried	42.82 (14.88)*	34.67 (17.58)*	5.96 (0.85)*	5.43 (1.07)*	2.68 (3.72)
Married	36.96 (14.39)	28.10 (14.96)	5.49 (0.97)	4.97 (1.08)	2.64 (3.29)
Exercise (times/week)					
≥ 3	38.07 (15.68)	29.52 (16.96)	5.55 (0.98)	5.02 (1.13)	2.56 (2.50)
< 3	38.09 (13.70)	29.20 (14.50)	5.60 (0.95)	5.09 (1.06)	2.72 (3.99)
BMI					
< 25	38.20 (14.08)	29.71 (14.91)	5.60 (0.95)	5.09 (1.07)	2.59 (3.21)
≥ 25	37.90 (15.49)	28.83 (16.80)	5.55 (0.98)	5.00 (1.13)	2.73 (3.61)

* : *p* < 0.05

For the analysis on the heart rate variability associated with job stress, the relationship with the heart rate variability in each subscale of KOSS was analyzed. In the group of high stress, SDNN was decreased in the area of occupational climate (*p* = 0.022), RMSSD was decreased in the area of job demand (*p* = 0.019), lack of reward (*p* = 0.033), occupational climate (*p* = 0.005), Hf(ln) was decreased in the area of organization system (*p* = 0.028), lack of reward (*p* = 0.039), occupational climate (*p* = 0.011) and LF/HF ratio was increased in the lack of reward area (*p* = 0.007) (Table 3).

**Table 3:** Mean values of heart rate variability by job stress

					Unit; Mean (S.D.)
	Time domain		Frequency domain		
Variables	SDNN	RMSSD	LF(ln)	HF(ln)	LF/HF ratio
Physical environment					
Low	37.47 (13.42)	28.91 (15.18)	5.55 (0.90)	5.05 (1.02)	2.53 (3.10)
High	38.62 (15.65)	29.75 (16.13)	5.60 (1.01)	5.06 (1.16)	2.74 (3.59)
Job demand					
Low	38.61 (14.73)	30.96 (16.41)*	5.59 (0.99)	5.13 (1.11)	2.39 (2.83)
High	37.64 (14.59)	28.04 (14.97)	5.58 (0.95)	5.00 (1.08)	2.85 (3.75)
Insufficient job control					
Low	38.40 (15.82)	29.79 (17.59)	5.59 (0.97)	5.09 (1.12)	2.49 (2.59)
High	37.84 (13.70)	29.02 (14.08)	5.57 (0.96)	5.03 (1.08)	2.77 (3.87)
Interpersonal conflict					
Low	37.92 (14.27)	27.86 (13.66)	5.62 (0.95)	4.97 (1.10)	3.04 (3.21)
High	38.09 (14.69)	29.46 (15.83)	5.58 (0.97)	5.06 (1.10)	2.62 (3.38)
Job insecurity					
Low	38.85 (14.78)	30.21 (16.81)	5.68 (0.94)*	5.10 (1.09)	2.85 (3.57)
High	37.39 (14.53)	28.59 (14.60)	5.49 (0.98)	5.02 (1.10)	2.46 (3.18)
Organizational system					
Low	38.84 (14.81)	30.52 (16.92)	5.61 (0.90)	5.17 (1.04)*	2.43 (3.00)
High	37.56 (14.54)	28.56 (14.76)	5.56 (1.01)	4.98 (1.13)	2.79 (3.60)
Lack of reward					
Low	39.40 (15.75)	31.26 (17.61)*	5.60 (0.94)	5.19 (1.08)*	2.21 (2.15)*
High	37.45 (14.08)	28.45 (14.62)	5.57 (0.98)	5.00 (1.10)	2.85 (3.80)
Occupational climate					
Low	39.54 (15.25)*	31.28 (17.50)*	5.69 (0.93)	5.18 (1.07)*	2.55 (3.13)
High	36.87 (14.05)	27.76 (13.84)	5.49 (0.98)	4.96 (1.11)	2.72 (3.57)
Total					
Low	38.51 (15.06)	30.41 (17.28)	5.60 (0.96)	5.12 (1.10)	2.54 (3.14)
High	37.65 (14.24)	28.30 (13.89)	5.56 (0.97)	5.00 (1.09)	2.75 (3.59)

* : *p* < 0.05

As the results of analysis on the relationships with the heart rate variability adjust for sociodemographic and job characteristics, SDNN and RMSSD were decreased in the area of occupational climate of the group with high job stress (*p* = 0.027, *p* = 0.036). HF(ln) was decreased in the area of organization system (*p* = 0.034) and occupational climate (*p* = 0.043) of the group with high stress. It was divided into groups by time and frequency domain (Tables 4 and 5).

**Table 4:** Analysis of covariance in KOSS and HRV (Time domain)^a^

		Unit; Mean (S.D.)
Variables	SDNN	RMSSD
Physical environment		
Low	37.47 (13.42)	28.91 (15.18)
High	38.62 (15.65)	29.75 (16.13)
Job demand		
Low	38.61 (14.73)	30.96 (16.41)
High	37.64 (14.59)	28.04 (14.97)
Insufficient job control		
Low	38.40 (15.82)	29.79 (17.59)
High	37.84 (13.70)	29.02 (14.08)
Interpersonal conflict		
Low	37.92 (14.27)	27.86 (13.66)
High	38.09 (14.69)	29.46 (15.83)
Job insecurity		
Low	38.85 (14.78)	30.21 (16.81)
High	37.39 (14.53)	28.59 (14.60)
Organizational system		
Low	38.84 (14.81)	30.52 (16.92)
High	37.56 (14.54)	28.56 (14.76)
Lack of reward		
Low	39.40 (15.75)	31.26 (17.61)
High	37.45 (14.08)	28.45 (14.62)
Occupational climate		
Low	39.54 (15.25)^*^	31.28 (17.50)^*^
High	36.87 (14.05)	27.76 (13.84)
Total		
Low	38.51 (15.06)	30.41 (17.28)
High	37.65 (14.24)	28.30 (13.89)

^a^Model was adjusted for age, smoking, alcohol intake, shift work, marital status and department

* : *p* < 0.05

**Table 5:** Analysis of covariance in KOSS and HRV (Frequency domain)^a^

			Unit; Mean (S.D.)
Variables	LF(ln)	HF(ln)	LF/HF ratio
Physical environment			
Low	5.55 (0.90)	5.05 (1.02)	2.53 (3.10)
High	5.60 (1.01)	5.06 (1.16)	2.74 (3.59)
Job demand			
Low	5.59 (0.99)	5.13 (1.11)	2.39 (2.83)
High	5.58 (0.95)	5.00 (1.08)	2.85 (3.75)
Insufficient job control			
Low	5.59 (0.97)	5.09 (1.12)	2.49 (2.59)
High	5.57 (0.96)	5.03 (1.08)	2.77 (3.87)
Interpersonal conflict			
Low	5.62 (0.95)	4.97 (1.10)	3.04 (3.21)
High	5.58 (0.97)	5.06 (1.10)	2.62 (3.38)
Job insecurity			
Low	5.68 (0.94)	5.10 (1.09)	2.85 (3.57)
High	5.49 (0.98)	5.02 (1.10)	2.46 (3.18)
Organizational system			
Low	5.61 (0.90)	5.17 (1.04)^*^	2.43 (3.00)
High	5.56 (1.01)	4.98 (1.13)	2.79 (3.60)
Lack of reward			
Low	5.60 (0.94)	5.19 (1.08)	2.21 (2.15)
High	5.57 (0.98)	5.00 (1.10)	2.85 (3.80)
Occupational climate			
Low	5.69 (0.93)	5.18 (1.07)^*^	2.55 (3.13)
High	5.49 (0.98)	4.96 (1.11)	2.72 (3.57)
Total			
Low	5.60 (0.96)	5.12 (1.10)	2.54 (3.14)
High	5.56 (0.97)	5.00 (1.09)	2.75 (3.59)

^a^Model was adjusted for age, smoking, alcohol intake, shift work, marital status and department

* : *p* < 0.05

## Discussion

We identified factors affecting the heart rate variability targeting firefighters and analyzed the relationships with job stress. The group with high job stress showed more decrease in the heart rate variability in the areas of occupational climate and organization system among subscales of KOSS compared to the group without it.

These results showed that continuous stress situations caused the sympathetic nervous acceleration and affected the development of cardiovascular diseases caused by increases in vascular resistance, vessel wall thickening, and induction of the metabolic syndrome and hypertension through various pathway. As the results of suppression of chronic parasympathetic nervous system, the chronic autonomous nervous system was deteriorated and inadequate responses to acute stress were produced. Thus, the physiological system were disturbed and the capability to maintain the homeostasis of the autonomous nervous system which was the defense mechanism was gradually degraded upon repetitive exposure to acute stress. It was consistent with the results of existing studies [8, 22]. In addition, the results of this study showed that the heart rate variability was determined by the control of the autonomous nervous system. Reductions of the heart rate variability caused by the sympathetic nervous acceleration or degradation of the parasympathetic nervous system directly or indirectly affected the heart. We tried to reveal that the reduction of the heart rate variability caused by stress was associated with cardiovascular diseases through these pathways. Moreover, since we selected firefighters that were shown to be vulnerable to cardiovascular disease in the previous study, our study is significant.

Heart rate variability is associated with general characteristics and lifestyle. In previous studies, Reardon et al. reported activities of the sympathetic nervous system were decreased with increasing age [23]. Umetani et al. reported that indicators of time domain were decreased with increasing age [24]. According to the results of this study, time domain (SDNN, RMSSD) and frequency domain (LF(ln), HF(ln)) were decreased with increasing age. It was consistent with results of previous studies.

Previous studies reported that the increases in LF/HF ratio were caused by acute effects of smoking [25]. The study targeting office workers showed that SDNN, LF and HF were decreased in the group of smoking [26]. In this study, LF(ln) was increased in the group of smoking, but RMSSD was also increased. The reason could be that we considered the status of currently smoking and we did not specify the differences in group distributions by age, smoking status right before the experiment and amount of smoking. As a result, we did not consider the acute effects and cumulative effects of smoking which may affect the heart rate variability. These points should be improved in the subsequent studies. The relationship between drinking and heart rate variability has been variously discussed. Previous studies reported that SDNN, LF and HF were decreased in the group of highly drinking such as 224 g in a week or drinking for 5 days in a week [26, 27]. Quintana et al. reported that the group of habitual drinkers showed the increases in HF compared to that of non-habitual drinkers [28]. It suggested appropriate amount of drinking reduced the incidence of cardiovascular diseases. In this study, SDNN, RMSSD and LF(ln) were significantly increased in the group of drinking compared to those of the group of non-drinking. It was thought that appropriate amount of drinking positively affected the activities of the autonomic nervous system. According to the results of existing studies, it has been known that exercise status and BMI are associated with the heart rate variability. However, in this study, there was no significant change. A previous study reported that appropriate exercise was increasing HF and decreases in SDNN, LF and HF were caused by lack of exercise [26, 29]. Other studies reported that activities of the parasympathetic nervous system were decreased by obesity, which decreased the heart rate variability. After the program for weight loss was performed, LF was decreased but HF and total power were increased in comparison with baseline [30, 31]. In this study, the groups of obesity were not evenly distributed because subjects with high obesity of BMI 30 or higher were few in number. Thus, it might affect the results. In the subsequent studies, we should specify the groups of exercising and non-exercising. Marital status and heart rate variability were related to age. Mean age of married group is higher than unmarried group. For that reason, heart rate variability was decreased in married group compared to unmarried group. Shift work is factor that affect heart rate variability in previous studies. Choi et al. reported very long (>48 h) shifts in firefighters reduced heart rate variability [32]. Amelsvoort et al. reported that decreased SDNN level during sleep in shift workers compared with day workers indicated a less favourable cardiovascular autonomic regulation, which may explain in part the excess cardiovascular disease risk in shift workers [33]. But, our study represent that RMSSD was increased in shift work group compared with non-shifting work group. Probably, these result was realted to age in common with results of marital status. In case of department, there were no stastical significance of heart rate variability to alalysis 4 departments (fire extinguish, rescue, emergency medical service, administration). RMSSD was decreased (*p* = 0.004) in the group of the administrative job that divided into the two groups of non-administrative job (fire extinguish, rescue, emergency medical service) and administrative job (administration). These results were realted to high mean age of administrative job compared with that of non-administrative job, too.

Many studies have been performed on the relationships between job stress and cardiovascular diseases on the basis of the job demand-control model of Karasek [34]. According to a review paper of Belkic et al., job stress was reported to be a major factor of cardiovascular diseases [35]. According to the study on the relationship between job stress and heart rate variability known as one of indicators of cardiovascular diseases, Togo et al. reported various job stress factors were associated with reductions of the heart rate variability [36]. It was reveled that job stress was associated with hyperactivity of the sympathetic nerves and reduced activities of parasympathetic nerves. In addition, the study targeting men workers at age of 40 or high in the shipyard showed that SDNN was significantly decreased in the group of high stress [37]. Hall et al. reported that the sleep disorder was caused by job stress and the heart rate variability was decreased by imbalance of the autonomic nervous system [38]. According to the study describing the relationship between job stress and heart rate variability using KOSS developed as a tool to measure job stress of Korean workers, it was reported that chronic autonomic nervous system was degraded in the group with high interpersonal conflict in the automobile manufacturer [39]. The study targeting men workers at age of 40 or high in the manufacturer showed that SDNN was significantly decreased in areas of job demand, job insecurity and interpersonal conflicts in the group of high job stress [8]. The study associated with area of lack of reward, Garza et al. reported that effort-reward imbalance was associated with decreases in SDNN, RMSSD and HF and increases in LF/HF ratio [40]. And study of Kim et al. reporting that monthly income was one of factors affecting psychosocial stress and fatigue of firefighters [41].

In the area of occupational climate, SDNN, RMSSD and Hf(ln) were decreased in the group of high stress. Unlike the reasonable workplace culture of the Western countries, these reductions were caused by authoritative and vertical workplace atmosphere, Korea’s unique collective culture such as office dinners or drinking culture and job conflicts such as random or inconsistent work orders. In addition, because emergency situations frequently occurred, firefighters were always tense and waiting, and they had irregular working hours and dangerous working environment. Therefore, vertical and rigid workplace cultures were partially associated with the particularity of firefighters [20]. According to the follow-up study performed by Hwang et al. using KOSS, it was reported that the relative risks of cerebrovascular diseases were 2.4 times higher in the area of occupational climate of the group with high job stress than those in the group with low job stress [42]. In the area of organization system, Hf(ln) was decreased in the group of high stress. Koh et al, reported that unreasonable organizational system was associated with increased total cholesterol level and PWV (pulse wave velocity) and decreased heart rate variability in time domain area [8]. It is also related to the particularity of firefighters such as lack of reasonable communication and organizational support to perform their duty.

This study has some limitations. First, this study was cross-sectional; therefore, we could identify the relationship of factors affecting the heart rate variability, but we did not clearly disclose the causality between them. The effect of long-term exposure to the job stress was not considered in this study, because we considered the levels of job stress measured at a certain time point upon inspection of the relationships with cardiovascular diseases. In the future, it should be considered through prospective study to provide supplementary data. Second, because the subjects of this study were firefighters in four fire stations located in a metropolitan city and they consisted of men, it was difficult to make comparison between men and women, generalize it for the entire firefighters or apply it to the general workers. In the future, the study should be performed with consideration of nationwide samples of firefighters, distribution of gender and various occupations. Third, the firefighters were exposed to high temperature and hazardous substances, because they frequently went to extinguish a fire. Carbon monoxide was also known as one of these hazardous substances. Davutoglu et al. reported that high levels of COHb caused by chronic exposure to carbon monoxide were associated with increases in hs-CRP which played an important role in atherosclerosis [43]. Bortkiewicz et al. presented the results in which carbon disulfide generated by a fire was also associated with reductions of the heart rate variability [44]. According to the study targeting employees in manufacturers, Son et al. reported that working environment with high temperature was associated with reductions of RMSSD [39]. This study included some questions about frequency of mobilization to a fire and wearing protective equipment for the last 1 year. However, most firefighters for on-site jobs did not remember the exact frequency of mobilization for the last 1 year upon consultation, and therefore it was actually difficult to set the levels of hazardous substances to which firefighters were exposed when they extinguished a fire. In addition, because there were high correlations between smoking and COHb, it was difficult to measure and analyze the heart rate variability in this study considering only exposure to hazardous substances. Nevertheless, this study analyzed factors that affect heart rate variability among firefighters in terms of their physical, occupational, socio-demographic and occupational stress. We also used KOSS which is reliable and valid measures to study the relationships between heart rate variability and cardiovascular diseases.

## Conclusion

In conclusion, we showed the changes in the heart rate variability depending on levels of job stress in the group of firefighters. In particular, the group with high stress showed significant reductions of the heart rate variability in areas of occupational climate and organizational system. With consideration of levels of job, it is necessary to improve Korea’s unique authoritative and vertical workplace culture in order to let individuals receive the trust and respect in the workplace. Also, reasonable communication in the workplace and organizational support to perform their duty were required by various ways. It is required to observe the patterns of changes through continuous follow-up studies on items of job stress in the future. Furthermore, it have to seek preventive medical care plans for analysis about the relationships with cardiovascular diseases and early detection of diseases through the analysis and evaluation of job stress factors. In addition, because this study is cross-sectional; we propose the time series studies on the relationships between job stress, heart rate variability and cardiovascular diseases through long-term prospective study.
